# Process Improvement Initiative to Reduce Average Length of Stay in a Community Hospital – A Preliminary Report

**DOI:** 10.46804/2641-2225.1197

**Published:** 2024

**Authors:** Alexander M. Reppond, Nicholas Flavin, Michael N. Albaum

**Affiliations:** a Department of Operations Transformation, MaineHealth, Portland, Maine; b Department of Quality Reporting & Data Analysis, MaineHealth, Portland, Maine; c Department of Medicine, MaineHealth, Portland, Maine

**Keywords:** Length of stay, Hospitals, Community hospitals, Quality improvement

## Abstract

**Introduction:**

Average length of stay (ALOS) has increased in many US hospitals in the post-COVID-19-pandemic world. We undertook a process improvement initiative to reduce the ALOS in our community hospital.

**Methods:**

Three core tactics were developed with a goal of reducing our ALOS by 10%. These tactics were early mobilization, Interprofessional Partnership to Advance Care and Education rounding, and structured interdisciplinary care rounds. Workgroups in each of these domains designed the improvement, devised measures of success, and implemented the tactic. A process improvement specialist worked with each workgroup using elements of the Model for Improvement. Process measures were reported weekly. Outcome measures (ALOS, observed vs expected LOS) were reported weekly. A central steering committee oversaw the initiative. All tactics were fully implemented by February 2023.

**Results:**

For the first 6 months after implementing our tactics, the ALOS on our inpatient medical units decreased from 6.3 to 5.5 days (13.7%) when compared with the same 6-month period in the prior year (P < .01).

**Discussion:**

We used 3 interventions to impact the ALOS in our community hospital. Preliminary data show a significant improvement. We cannot isolate the independent contribution of each intervention and did not control for confounders.

**Conclusions:**

Our interdisciplinary team developed and implemented tactics to reduce the ALOS in our community hospital by 13.7%.

## 1. Introduction

Since the COVID-19 pandemic began, the average length of stay (ALOS) for inpatients has significantly increased.^1^ Longer ALOS can have several deleterious effects on patients and hospital operations,^2^ including negatively impacting patient experience^3^ and increasing the risk for hospital acquired conditions.^4^ Hospitalization beyond what is medically necessary also results in overcrowding of inpatient units, backing up of patients awaiting admission in the emergency department, and staff burnout.^2,5,6^ There are financial implications as well because hospitals are paid, in most cases, with a fixed Diagnosis Related Group (DRG) payment based on the principal diagnosis.^7^ The cost of hospitalization after the expected discharge may not be reimbursed, adding to financial pressure on the organization.

At our community hospital in Southern Maine, inpatient ALOS remained elevated post-pandemic, both in absolute terms and on a risk-adjusted basis. We undertook a process improvement initiative to reduce ALOS on our adult medicine units. Here we report on the process improvement methods used and their preliminary impact on ALOS.

### 1.1. Background

The setting of the initiative was Southern Maine Health Care, a community hospital in Biddeford, Maine, with 161 total beds, of which 103 are adult medicine beds. These beds are predominantly staffed by our hospitalist service.

During the pandemic, inpatient ALOS at Southern Maine Health Care increased significantly. Factors contributing to this increase included lower capacity at skilled nursing facilities (SNFs) due to staffing issues, extended hospital stays required by SNFs due to new policies to prevent infection, and greater reliance on contracted nursing and physician staff. The inpatient ALOS increased from 4.45 days before the pandemic to more than 7 days during the pandemic.

Despite being more than 2 years into the post-pandemic world, the longer ALOS did not return to baseline. Potential contributors to this longer ALOS included continued reliance on contract labor, persistent staffing constraints at SNFs, delays in transportation services, lower capacity of home care agencies, and reduced hospital efficiency due to a high census.

To address the problem, we assembled a multidisciplinary team to explore solutions. This team included leadership from hospital medicine, nursing, case management, and physical therapy, as well as program management and data analysis members of our quality department. Recognizing the need to return to basic principles of inpatient care, the team identified 3 core strategies to address ALOS, all of which centered around enhancing interprofessional communication and practice through structure.

### 1.2. Early mobilization

This intervention was based on work at Johns Hopkins Medicine, where mobilizing patients early and often reduced ALOS.^8^ Although the expectation of mobilizing patients was in place before this intervention, there was no structure to assess progress.

### 1.3. Adoption of interprofessional rounding

Previous studies indicated that having multiple disciplines round simultaneously at the patient’s bedside can improve interprofessional communication, enhance coordination of care, and reduce LOS. The Interprofessional Partnership to Advance Care and Education (iPACE) model showed improved team functioning and professional experience, as well as positive patient feedback, when piloted on an inpatient medicine unit at Maine Medical Center, our health system’s tertiary care and principal academic center.^9^ The structured interprofessional communication was particularly important to us because of our heavy reliance on contracted nursing and physician staff, whom may not be familiar with one another. The iPACE pilot unit also showed substantial cost savings due to a shorter ALOS of 0.74 days compared with a similar unit when averaged across similar DRGs and averaged over 2 years.^10^ Before the implementation of this initiative, interprofessional bedside rounding was not a core expectation, occurred infrequently, and was unstructured.

### 1.4. Enhanced discharge planning (interdisciplinary care rounds)

Although this process was in place before our process improvement initiative, it was not structured nor consistently facilitated. Therefore, our initiative focused on adding that structure. Also, this initiative added an interdisciplinary “escalation huddle” in the midafternoon to improve communication and escalate any barriers to patient discharge.

### 1.5. Purpose and goals

Our quality improvement initiative aimed to implement process changes in the 3 core strategies outlined above and to evaluate their effectiveness in reducing ALOS.

## 2. Methods

### 2.1. Patient population

The patient population studied included hospitalized inpatients on our hospitalist-staffed adult medicine service, which included most of our surgical and orthopedic admissions as well. Newborns, maternity, and admissions to our behavioral health unit were not included in the interventions or analysis. We also excluded extreme “long stay” patients with a hospital stay greater than 180 days to reduce skewing of data.

### 2.2. Early mobility

At the time of admission, patients’ baseline mobility was assessed using a validated tool: the Bedside Mobility Assessment Tool (BMAT).^11^ The results were documented in the electronic health record (EHR; Epic). Patients who ambulate independently (BMAT 4) or with minimal assistance (BMAT 3) were given a daily mobility goal, documented on the bedside whiteboard. Physical therapy (PT) assisted nursing with training on the tool, assignment of goals, and mobilization. However, nursing was primarily responsible for encouraging patients and tracking progress toward the mobility goal. This intervention started on February 6, 2023.

Data were collected via twice weekly audits conducted by nursing managers and directors. These data specifically included whether a mobility goal was documented on the patient’s whiteboard and whether progress toward the goal was documented in the EHR.

### 2.3. IPACE bedside rounding

We defined iPACE bedside rounding as a provider (physician or advanced practice provider) and the primary nurse rounding together at the bedside in the morning. To facilitate the rounding, it was necessary to alter provider and nursing assignments to groups of patients who were geographically cohorted. Optimally, a provider with a panel of 15 patients would round with 3 nurses (who were each assigned 5 patients). It was not always possible to make this happen, but the model worked best the closer we got to that ideal workflow. Each of the 7 rounding teams were assigned a case manager, who interacted with the provider and charge nurses. This intervention started on February 14, 2023.

Provider and nurse rounding adherence were collected by self-report from the floor nurses to the charge nurse. Staff from our patient experience department performed spot checks, asking patients if they experienced iPACE rounds and if core elements (medications, discharge plans) were addressed.

### 2.4. Interdisciplinary care round optimization

Daily interdisciplinary care rounds (IDCR) took place in a conference room separate from the bedside before the other interventions. The meeting was run by our case management, with participation by all allied health professionals, including PT, occupational therapy, dietary, pharmacy, respiratory therapy, nursing, and providers. Two separate rounds took place, one for each of our adult medicine floors.

To improve efficiency, we stopped requiring providers to attend IDCR and had the case managers bring all relevant information from their assigned team. At the IDCR, allied health professionals worked to identify and remove barriers to discharge. This approach permitted providers to focus on discharges before noon.

A process change to IDCR was focusing on the estimated date of discharge. This date, available in our EHR, is the predicted geometric mean length of stay (GMLOS) associated with the principal diagnosis as published by the Centers for Medicare and Medicaid Services. This date is entered into the EHR for each patient admitted to the hospital. Barriers to discharge by that date were discussed and mitigated. This process and structure change started on February 6, 2023.

A consultant on site assisted with the ALOS improvement (Claro Healthcare) and aided in developing an audit tool for the effectiveness of the IDCR. They also suggested an enhancement, which we subsequently implemented, to the IDCR consisting of a midafternoon virtual huddle to identify any lingering barriers to the day’s discharges. That huddle involved nursing leadership, case management, physical therapy, occupational therapy, and physician leadership.

### 2.5. Average length of stay

For this analysis, ALOS represents the total number of days for an inpatient episode for our study population, calculated by subtracting the date of admission from the date of discharge. To account for the risk, we calculated the ratio of the observed ALOS to the expected LOS using the GMLOS for each patient’s Medicare Severity DRGs. This ratio is referred to as the GMLOS Index. We compared ALOS and GMLOS Index data for the same 6-month period (March through August) in both 2022 and 2023 to mitigate seasonal variations. Although the day of admission was counted in computing the number of discharge days, the day of discharge was not.

### 2.6. Project management and communication

Separate workgroups planned and implemented each of our 3 principal initiatives. Also, a data/analytics workgroup assembled and presented data, and a steering committee oversaw the project. Using the Model for Improvement Framework,^12^ project management deployed multiple rounds of Plan/Do/Study/Act cycles within each of the 3 initiatives. A consultant (Claro Healthcare) had been engaged to help our organization address ALOS. The initiatives had already been chosen before the consultant arrived, but their work validated our approaches.

The project launch was communicated to staff through their staff meetings and via email newsletter. Process and outcome measures were reviewed weekly at our facility’s daily operations huddle. Senior leadership was kept updated on the status of the project.

The MaineHealth Institutional Review Board provided a “not research” determination.

### 2.7. Statistical analysis

The pre-intervention and post-intervention study periods were defined as any qualifying patient discharge between March 1 and August 31 in 2022 and 2023. We performed a 2-tailed Mann-Whitney U test to compare changes in patient ALOS and an unpaired *t*-test to compare changes in patient GMLOS Index between the pre-intervention and post-intervention periods. Results were considered significant with a *P* ≤ .05. Outliers were detected using Tukey’s fence method (k = 1.5) and were included in the analysis. Data were aggregated for presentation by month and over each 6-month study period.^13^

Summary and inferential statistics were calculated using both Microsoft Excel 365 (version 2402) and Dotmatics GraphPad Software (GraphPad Prism version 10 for Windows, GraphPad Software, www.graphpad.com). Tableau Software (version 9.2) was used to visualize the data, including adding summary statistics (eg, means, standard deviations) to the figures.

## 3. Results

**Fig. 1.**
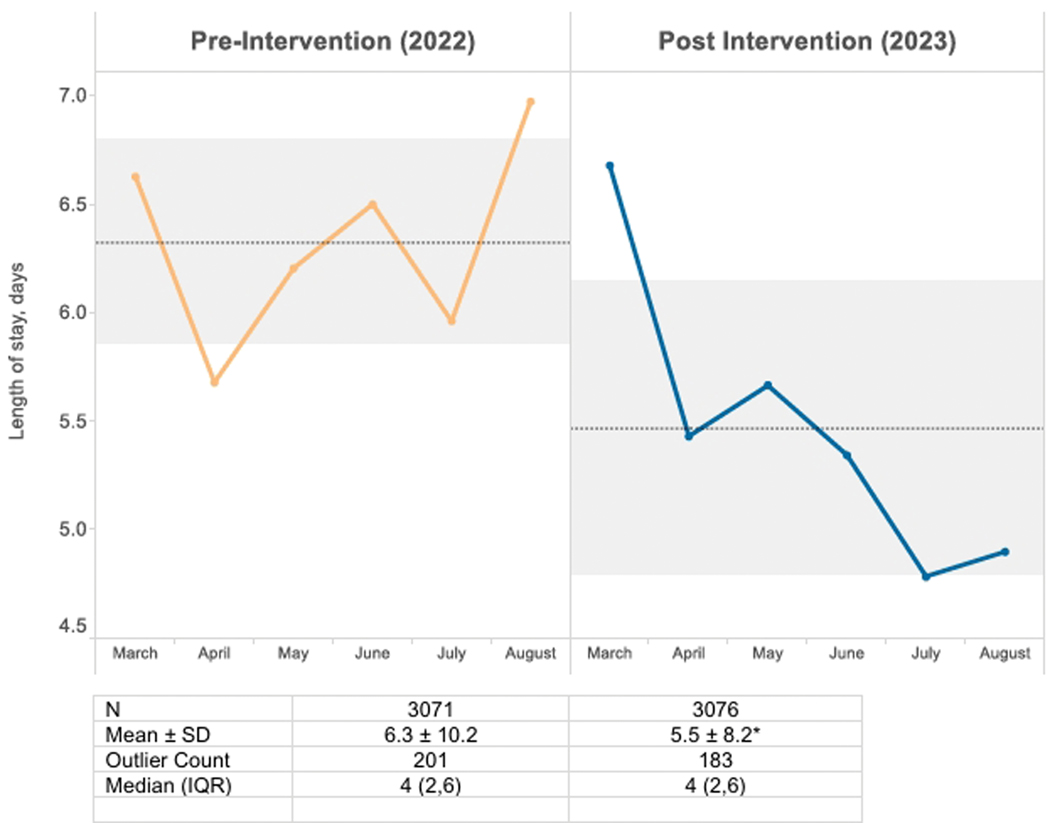
Average Inpatient Length of Stay for Patients Discharged Between March 1 and August 31 in 2022 (Pre-Intervention) and 2023 (Post-Intervention). Gray bands represent mean ± SD. *P≤ 0.001 vs the pre-intervention period. IQR, interquartile range.

A total of 12197 inpatient discharges were evaluated between March 1 and August 31 in 2022 and 2023 (Fig. 1). Patient ALOS increased to an average of 6.3 days from the baseline (2022). Post-implementation (2023), ALOS decreased from 6.3 to 5.5 days, an absolute reduction of 0.9 days or a 13.7% relative reduction (P < .05). The median and interquartile range for the pre-intervention and post-intervention periods were the same, but we observed fewer outliers. The GMLOS Index also decreased between the pre-intervention and post-intervention periods (95% CI, 0.023–0.172; P < .05; data not shown).

Fig. 1 summarizes monthly ALOS data for March to August in 2022 and 2023, and shows reduced ALOS after full implementation of our 3 interventions in February 2023.

**Fig. 2.**
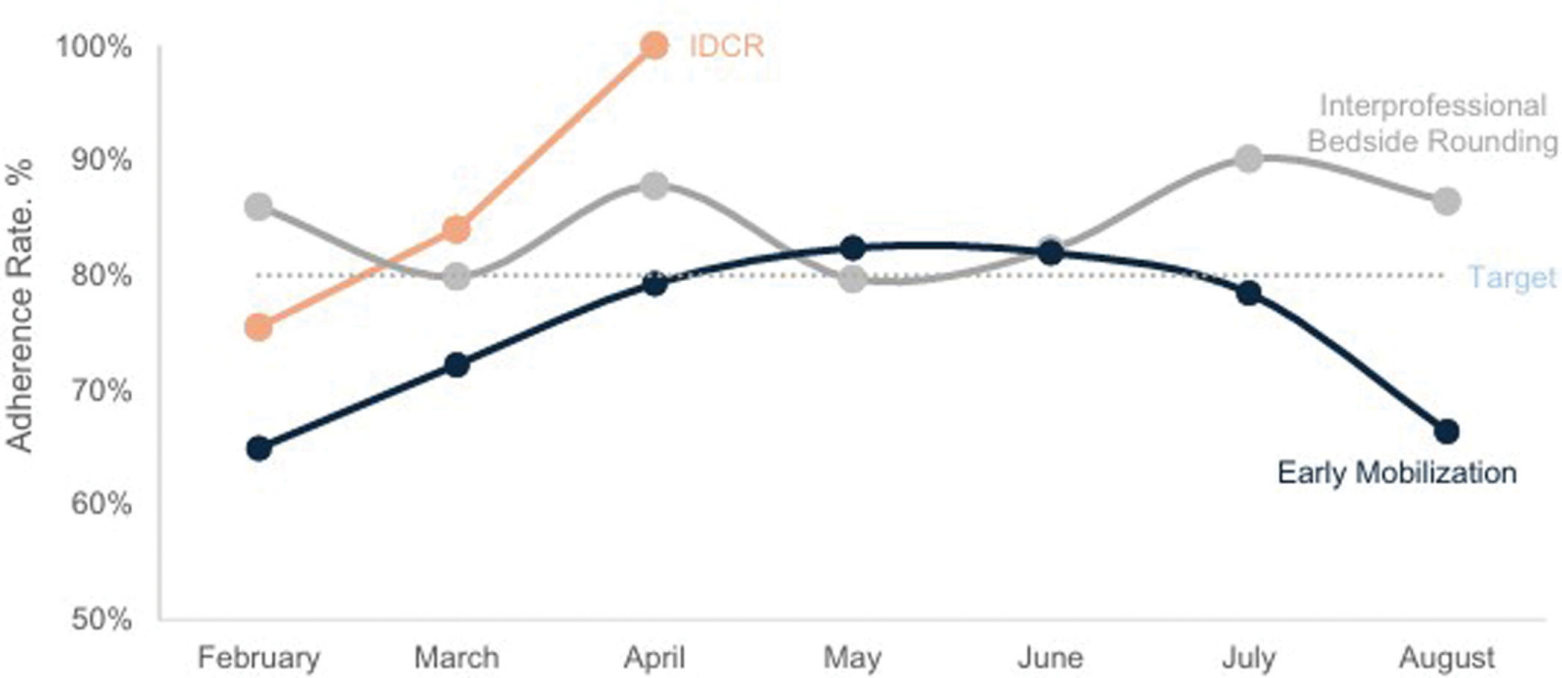
Rates of Adherence for three Interventions from February to August 2023. IDCR, interdisciplinary care rounds.

Fig. 2 shows intervention adherence rates during the post-intervention period. Although the IDCR continued during the entire intervention period, given the high reliability documented during 3 months of audits, we stopped the audits after 3 months. Both IDCR and iPACE bedside rounding consistently met or exceeded our pre-intervention target of 80% adherence. Early mobilization did not consistently meet that percentage.

## 4. Discussion

We used a multimodal intervention to reduce the ALOS in our community hospital, and preliminary data suggest that our interventions were successful. Our process involved bringing together leadership from multiple disciplines to design the interventions and was informed by the knowledge and experience of our care team leads. We limited the project to interventions with evidence of impact on ALOS and included only 3 interventions, as this number was feasible to implement and monitor. As the interventions were implemented as a bundle, we were unable to determine the relative impact of each intervention on ALOS.

Using techniques from the Model for Improvement,^12^ we assigned a target of 80% reliability before deciding if we were meeting our overall goal to reduce ALOS. Though the 3 process measures did not all maintain that percentage, given the improved ALOS, we focused on sustaining the interventions rather than launching new initiatives, and we plan to continue with these efforts.

We appreciate that other factors may have contributed to the reduced ALOS. For example, improvements over time in access to SNFs, home health services, and transportation may have contributed to the observed decline. Also, we reduced our reliance on contract (travel) nursing services during the post-intervention period (data not shown), which also may have had an impact. Areas for future study include the impact of our interventions on other outcome measures, such as patient experience and any impact on hospital acquired conditions.

## 5. Conclusions

Our intervention bundle of interdisciplinary care, iPACE bedside rounding, and early mobilization was associated with a 13.7% reduction in ALOS. Although our process improvement initiative may not be generalizable to other institutions, this study shows that applying principles of improvement science can successfully improve processes affecting ALOS in a community hospital setting.
